# High-Dimensional ICA Analysis Detects Within-Network Functional Connectivity Damage of Default-Mode and Sensory-Motor Networks in Alzheimer’s Disease

**DOI:** 10.3389/fnhum.2015.00043

**Published:** 2015-02-03

**Authors:** Ottavia Dipasquale, Ludovica Griffanti, Mario Clerici, Raffaello Nemni, Giuseppe Baselli, Francesca Baglio

**Affiliations:** ^1^IRCCS, Don Gnocchi Foundation, Milan, Italy; ^2^Department of Electronics, Information and Bioengineering, Politecnico di Milano, Milan, Italy; ^3^FMRIB (Oxford University Centre for Functional MRI of the Brain), Oxford, UK; ^4^Università degli Studi di Milano, Milan, Italy

**Keywords:** group independent component analysis, functional connectivity, resting-state fMRI, Alzheimer’s disease, default-mode network, sensory-motor network

## Abstract

High-dimensional independent component analysis (ICA), compared to low-dimensional ICA, allows to conduct a detailed parcellation of the resting-state networks. The purpose of this study was to give further insight into functional connectivity (FC) in Alzheimer’s disease (AD) using high-dimensional ICA. For this reason, we performed both low- and high-dimensional ICA analyses of resting-state fMRI data of 20 healthy controls and 21 patients with AD, focusing on the primarily altered default-mode network (DMN) and exploring the sensory-motor network. As expected, results obtained at low dimensionality were in line with previous literature. Moreover, high-dimensional results allowed us to observe either the presence of within-network disconnections and FC damage confined to some of the resting-state subnetworks. Due to the higher sensitivity of the high-dimensional ICA analysis, our results suggest that high-dimensional decomposition in subnetworks is very promising to better localize FC alterations in AD and that FC damage is not confined to the DMN.

## Introduction

Independent component analysis (ICA) is a powerful data-driven method used for functional connectivity (FC) analysis of the resting-state fMRI (rfMRI) data. It decomposes rfMRI data into distinct networks, the resting-state networks (RSNs), correlated in their spontaneous fluctuations but also maximally independent in the spatial domain (Beckmann et al., 2005).

Current research in rfMRI is increasingly adopting group-level high-dimensional ICA to obtain more detailed and informative network analyses with respect to the more common low-dimensional approach (Kiviniemi et al., 2009; Abou-Elseoud et al., 2010; Abou Elseoud et al., 2011; Smith et al., 2011, 2013; Tian et al., 2013). In fact, the splitting of the RSNs, obtained from the high-dimensional ICA analysis, could be due to a differential functionality of subnetworks forming the larger ones obtained with the low-dimensional analysis (Smith et al., 2009; Abou-Elseoud et al., 2010). In applications to pathological conditions, this differential functionality of subnetworks could be related to the specific set of subjects (Abou-Elseoud et al., 2010; Damoiseaux et al., 2012) and driven by the pathology itself, allowing a more disease-specific FC analysis. Abou-Elseoud et al. (2010) showed that ICA analysis results are affected by model order selection and demonstrated by patients with seasonal affective disorder (Abou Elseoud et al., 2011) that the between-group differences measured with ICA increase with model order (reaching a maximum of around 70 components on data acquired with a standard EPI sequence), thus suggesting multi-level ICA exploration of RSNs FC to optimize sensitivity to brain disorders. Furthermore, the analysis of the temporal information obtained from rfMRI data (i.e., from the analysis of the time series associated with each component) with low- and high-dimensional ICA allows the study of brain function from a complementary perspective to the information provided by the spatial map analysis. This was also confirmed by Tian et al. (2013) in a recent study conducted on healthy subjects investigating the spatial and temporal features of rfMRI related to behavior, wherein they highlighted the benefit of the temporal analysis of the RSNs.

In this work, we applied these innovative rfMRI analyses to Alzheimer’s disease (AD), the most common cause of dementia in the elderly (Berr et al., 2005). In fact, the decreased FC of the default-mode network (DMN), quantified on rfMRI data, is becoming a possible new biomarker for this pathology (Greicius et al., 2004; Gili et al., 2011; Li and Wahlund, 2011). Therefore, early detection and a detailed characterization of this alteration are crucial. Moreover, recent rfMRI studies also investigated the effects of AD in other RSNs (Brier et al., 2012; Li et al., 2012; He et al., 2013) in order to investigate possible additional FC changes beyond the DMN and memory function, but the impact of the pathology on these networks is still unclear.

This study was led by the hypothesis that, in the first stages of AD, functional alterations arise as within-network FC loss, and, as the severity of illness increases, these alterations involve the whole brain. We used high-dimensional ICA to decompose the RSNs in subnetworks and to explore the within-network disruption mechanisms that affect the mild and moderate stages of AD. We also compared the results of this approach with the more traditional low-dimensional ICA one.

We first used an objective and automatic algorithm to associate the resting-state subnetworks obtained by means of the high-dimensional ICA (i.e., 70 components) to the major RSNs described in literature and obtained with the low-dimensional ICA (i.e., 25 components). We explored the FC in patients with AD and healthy controls (HC) within two RSNs, the DMN and the sensory-motor network (SMN). The first network, which comprises the medial prefrontal cortex (mPFC), the posterior cingulate cortex (PCC), the inferior parietal lobes, and the hippocampus, is the primarily altered RSN in AD, while the SMN is still poorly explored in AD studies. The low-dimensional spatial map analysis was used to verify that the results of our study were in line with previous literature (Greicius et al., 2004; Zhang et al., 2009; Binnewijzend et al., 2012; Hafkemeijer et al., 2012); then, by means of the temporal (amplitude and network) analysis at low dimensionality, and the spatial and temporal analyses at high dimensionality, we investigated the FC of the selected RSNs and their subnetworks in more detail.

## Material and Methods

### Subjects

Forty-one subjects (21 AD and 20 HC) participated in the study. Patients with AD [mean age 73.62 ± 5.22; 8 males] were recruited from the Memory Clinic of I.R.C.C.S. Don Gnocchi Foundation, with a diagnosis of probable AD dementia, according to the revised NINCDS-ADRDA criteria (McKhann et al., 2011), in a mild to moderate stage [Clinical Dementia Rating Scale (CDR) ≤ 2]. To increase the diagnostic accuracy, analyses of hippocampal volumes were also included in the study as an index of downstream neural injury according to the guidelines for Alzheimer’s dementia (McKhann et al., 2011). Twenty age-matched HC (mean age 71.05 ± 3.66; 7 males, MMSE ≥ 28) had no history of neurological, cardiovascular, or metabolic disorders and voluntarily participated in the study. According to the recommendations of the declaration of Helsinki for investigations on human subjects, both local ethics committee approval of the Don Gnocchi Foundation and written informed consent from all subjects or their caregivers to participate in the study were obtained before study initiation.

Subjects’ demographic details are reported in Table 1.

**Table 1:** **Demographical and anatomical information of the sample**.

	HC	AD	Group comparison (*p*-value)
*N*	20	21	
Age (years, mean ± SD)	71.05 ± 3.66	73.62 ± 5.22	0.08
Gender (F:M)	13:7	13:8	0.21
MMSE score (mean ± SD)	29.55 ± 0.69	21.62 ± 2.71	<0.01*
Right hippocampal volume (mm^3^, mean ± SD)	3746.7 ± 586.3	2837.9 ± 537.1	<0.05*
Left hippocampal volume (mm^3^, mean ± SD)	3594.4 ± 510.8	2678 ± 566.3	<0.05*
Motion during fMRI acquisition^a^	0.07 ± 0.04	0.09 ± 0.06	0.27

*HC, healthy controls; AD, Alzheimer’s disease; SD, standard deviation; MMSE, mini mental state evaluation*.

*The group comparison was calculated with two-sample independent *t*-test or Fisher’s exact test, as appropriate*.

*Significant (*p* < 0.05) compared to HC and AD group.

*^a^Mean relative displacement in mm as calculated during the pre-processing with MELODIC FSL tool*.

### MRI acquisitions and analyses

#### MRI acquisition protocol

Brain MR images were acquired using a 1.5 T scanner (Siemens Magnetom Avanto, Erlangen, Germany) with eight-channel head coil. rfMRI, BOLD EPI images (TR/TE = 2500/30 ms; resolution = 3.1 × 3.1 × 2.5 mm; matrix size = 64 × 64; number of axial slices = 39; number of volumes = 160; flip angle = 70°; acquisition time 6 min and 40 s) were collected at rest. Subjects were instructed to keep their eyes closed, not to think about anything in particular, and not to fall asleep. High-resolution T1-weighted 3D images (TR = 1900 ms; TE = 3.37 ms; matrix 192 × 256; resolution 1 × 1 × 1 mm^3^; 176 axial slices) were also acquired and used as anatomical references for fMRI analysis and for hippocampal volume calculation with FSL-FIRST tool (Patenaude et al., 2011).

#### rfMRI data analysis

Pre-processing of rfMRI data was carried out using FSL (Smith et al., 2004; Jenkinson et al., 2012). Standard pre-processing steps involved: motion correction, non-brain tissues removal, spatial smoothing with a 5 mm full width at half maximum Gaussian kernel, and high-pass temporal filtering with a cutoff frequency of 0.01 Hz. Subsequently, single-subject spatial ICA with automatic dimensionality estimation was performed using MELODIC (Beckmann and Smith, 2004) and FMRIB’s ICA-based Xnoiseifier (FIX, http://fsl.fmrib.ox.ac.uk/fsl/fslwiki/FIX) (Salimi-Khorshidi et al., 2014) was used to regress the full space of motion artifacts and noise components out of the data (Griffanti et al., 2014). The training set for FIX was obtained using a separate group of HC (*N* = 42; age 35.7 ± 22.3 years; M/F = 19/23). However, due to the modest number of subjects, we were also able to manually check that FIX successfully identified the artifactual components on data of patients with AD.

After the pre-processing, each single-subject 4D dataset was first aligned to the subject’s high-resolution T1-weighted image using linear registration (FLIRT – Jenkinson and Smith, 2001; Jenkinson et al., 2002) enhanced with brain-boundary registration (BBR – Greve and Fischl, 2009), then registered to MNI152 standard space using non-linear registration (FNIRT), and subsequently resampled to 2 × 2 × 2 mm^3^ resolution.

The rfMRI data were then temporally concatenated across subjects and group-ICA was performed using MELODIC. For the low-dimensional ICA, we chose a model order of 25 ICs, in line with previous studies and guidelines (Filippini et al., 2009; Smith et al., 2009; Abou-Elseoud et al., 2010; Damoiseaux et al., 2012; Zamboni et al., 2013). For the high-dimensional ICA, we chose a dimensionality of 70 ICs, as suggested in Abou-Elseoud et al. (2010) and Abou Elseoud et al. (2011) and judged to be compatible with the temporal degrees of freedom of the data (after the cleaning procedure). Subject-specific time series and spatial maps from the low-dimensional and high-dimensional group ICs were obtained with the dual-regression approach (Filippini et al., 2009).

The low-dimensional group ICs were manually classified as RSNs or artifacts based on previous knowledge of the RSN patterns described in literature (Beckmann et al., 2005; De Luca et al., 2006; Rytty et al., 2013). The high-dimensional components were then classified taking the low-dimensional RSNs as reference templates, and using a spatio-temporal labeling criterion: the high-dimensional component *i* was labeled as part of the low-dimensional component *j* with which it had the highest spatial overlap (calculated with Dice coefficient, DC*_ij_*) and the highest temporal correlation (TC*_ij_*, calculated with Pearson’s correlation among single-subject time series and averaged across subjects). A component was classified as residual noise if all DC*_ij_* or TC*_ij_* were below a threshold empirically determined during the algorithm development by evaluating different values against manual classification (namely 0.1 for DC*_ij_* and 0.4 for TC*_ij_*) on three different datasets, included the one used in this study, or classified as unknown if the spatial and temporal matching results disagreed. Both the residual noise and the unknown components were ignored in subsequent analyses.

Subsequent analyses were focused on the two RSNs of interest: the DMN and the SMN. We performed spatial maps and temporal (amplitude and network) analyses on these ICs of interest, both at low and high dimensionality. Regarding the time series analysis at high dimensionality, we will use the terms “within-network” and “between-network,” respectively, referring to comparisons made between subareas belonging to the same RSN or different RSNs.

The spatial maps derived for each subject from the second stage of dual regression were compared between the two groups through voxel-wise statistics using a non-parametric permutation test. A cluster-based thresholding was used, corrected for multiple comparisons by using the null distribution of the max cluster size (Hayasaka and Nichols, 2003).

From the subject-specific time series, obtained as output of the first stage of dual regression, we calculated the amplitude of the selected RSNs as the standard deviation of the time series. Network analysis was also performed by estimating full correlation values (then converted into *z*-values) between all pairs of time series of the selected components. Significant differences in amplitude and correlations between HC and patients with AD were then assessed with two-sample *t*-test and corrected for multiple comparisons using FSLNets (http://fsl.fmrib.ox.ac.uk/fsl/fslwiki/FSLNets).

## Results

### Demographical and anatomical characteristics of the participants

HC subjects and patients with AD were comparable for age and gender; a significant difference was found for MMSE score, accordingly with the adopted inclusion criteria (Table 1). The significant difference in hippocampal volumes confirmed the gray matter volume alteration typical of AD.

### Low- and high-dimensional ICA results

#### rfMRI components identification

**Figure 1:**
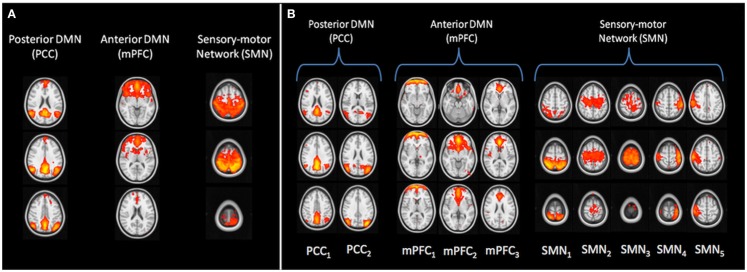
**Resting-state networks and corresponding subnetworks, revealed by low-dimensional group-ICA (A) and high-dimensional ICA (B)**. Images are shown in radiological orientation. DMN, default-mode network; SMN, sensory-motor network; PCC, posterior cingulate cortex; mPFC, medial prefrontal cortex.

Out of the 25 components detected by low-dimensional ICA, we identified 10 RSNs and focused our analysis on the posterior portion of the DMN, which included the PCC, the inferior parietal lobule and part of the frontal lobe (from now on, we will refer to this component as the PCC component), the anterior part of the DMN, mainly the mPFC, and the SMN (Figure 1A). The high-dimensional components belonging to the selected RSNs, according to the spatio-temporal labelling criterion, are shown in Figure 1B): the PCC was identified in two components (PCC_1_, PCC_2_), the mPFC in three components (mPFC_1_, mPFC_2_, and mPFC_3_), and five components (SMN_1_, SMN_2_, SMN_3_, SMN_4_ and SMN_5_) were recognized as belonging to the SMN.

#### Time series amplitude

As reported in Table 2, at low dimensionality, we observed that patients with AD showed significantly decreased amplitude values in the three selected components. The same analysis performed at high dimensionality revealed significant decreased amplitude in patients with AD in one of the two PCC subnetworks (PCC_1_) and in one component within the mPFC (the ventral mPFC, mPFC_2_). Results survived correction for multiple comparisons across components at low dimensionality and across subcomponents within each RSN at high dimensionality.

**Table 2:** **Time series amplitudes**.

*D* = 25				*D* = 70			
Component	HC	AD	*p*-value	Component	HC	AD	*p*-value
PCC	1.71 ± 0.33	1.36 ± 0.44	0.007^a^	PCC1	1.66 ± 0.29	1.36 ± 0.35	0.005^a^
				PCC_2_	1.46 ± 0.33	1.21 ± 0.39	0.03
mPFC	1.51 ± 0.38	1.18 ± 0.38	0.009^a^	mPFC1	1.14 ± 0.22	1.03 ± 0.22	n.s.
				mPFC_2_	1.24 ± 0.2	1.04 ± 0.24	0.007^a^
				mPFC3	1.22 ± 0.27	1.17 ± 0.22	n.s.
SMN	1.72 ± 0.59	1.33 ± 0.39	0.016^a^	SMN1	1.51 ± 0.43	1.36 ± 0.34	n.s.
				SMN_2_	1.47 ± 0.33	1.22 ± 0.25	0.009
				SMN3	1.5 ± 0.3	1.39 ± 0.23	n.s.
				SMN4	1.46 ± 0.42	1.27 ± 0.25	n.s.
				SMN5	1.35 ± 0.35	1.21 ± 0.33	n.s.

*Comparison between the two groups at low and high dimensionality (two-sample *t*-test)*.

*^a^Group differences surviving after correction for multiple comparisons across components (low dimensionality) and subcomponents (high dimensionality) within each RSN*.

#### Network analysis

At low dimensionality, only the between-network connectivity PCC–mPFC was significantly different between the two groups (mean *z*-score_HC_ = 5.65 ± 3.13, mean *z*-score_AD_ = 3.46 ± 2.91, *p*_corr_ = 0.03).

**Figure 2:**
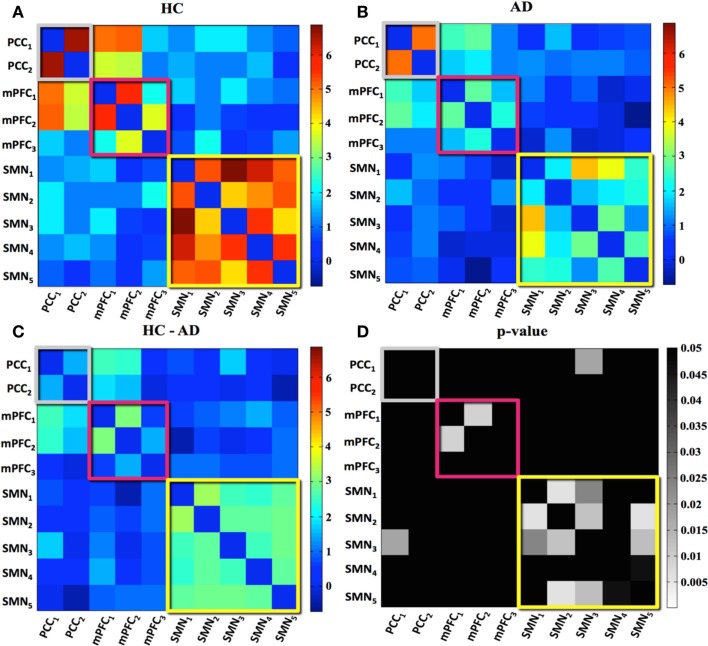
**Subnetwork correlation matrices for (A) healthy controls (HC) and (B) patients with Alzheimer’s disease (AD)**. Intra-network correlations appear on diagonal blocks; inter-network correlations appear in off diagonal blocks. **(C)** and **(D)**, respectively, show the HC–AD difference matrix and the significant differences in amplitude and correlations between HC and patients with AD, corrected for multiple comparisons. Colored boxes denote network membership: Gray – PCC; red – mPFC; yellow – SMN. The color bar indicates the range of correlation values (blue: *z*-score = –1; red: *z*-score = 7). The color bar in **(D)** indicates statistically significant *p*-values in grayscale, while *p*-values ≥ 0.05 are black colored.

Figure 2 summarizes high-dimensional results. FC is, respectively, expressed as the mean *z*-score across HC (Figure 2A) and patients with AD (Figure 2B). The within-network FC is arrayed in blocks along the diagonal and the between-network FC appears outside the blocks. Figure 2C, showing the differences of correlation values, highlights a generally reduced within-network connectivity and isolated loss of between-network connectivity in AD compared to HC (the warm hues indicate loss of FC in AD). Statistical results of FC decrease in AD (*p* < 0.05, FWE-corrected for multiple comparisons) are reported in Figure 2D.

With regard to the DMN, the within-network connectivity was not different in the PCC component, while FC alteration in AD was detected among one of the three connections in the mPFC network, namely those involving the ventral mPFC (mPFC_1_–mPFC_2_), in which we previously observed the amplitude decrease.

The same analysis in the SMN resulted in decreased within-network connectivity among all the subnetworks belonging to the SMN, and one altered between-networks connection between SMN and DMN (PCC_1_–SMN_3_).

#### Spatial maps analysis

**Figure 3:**
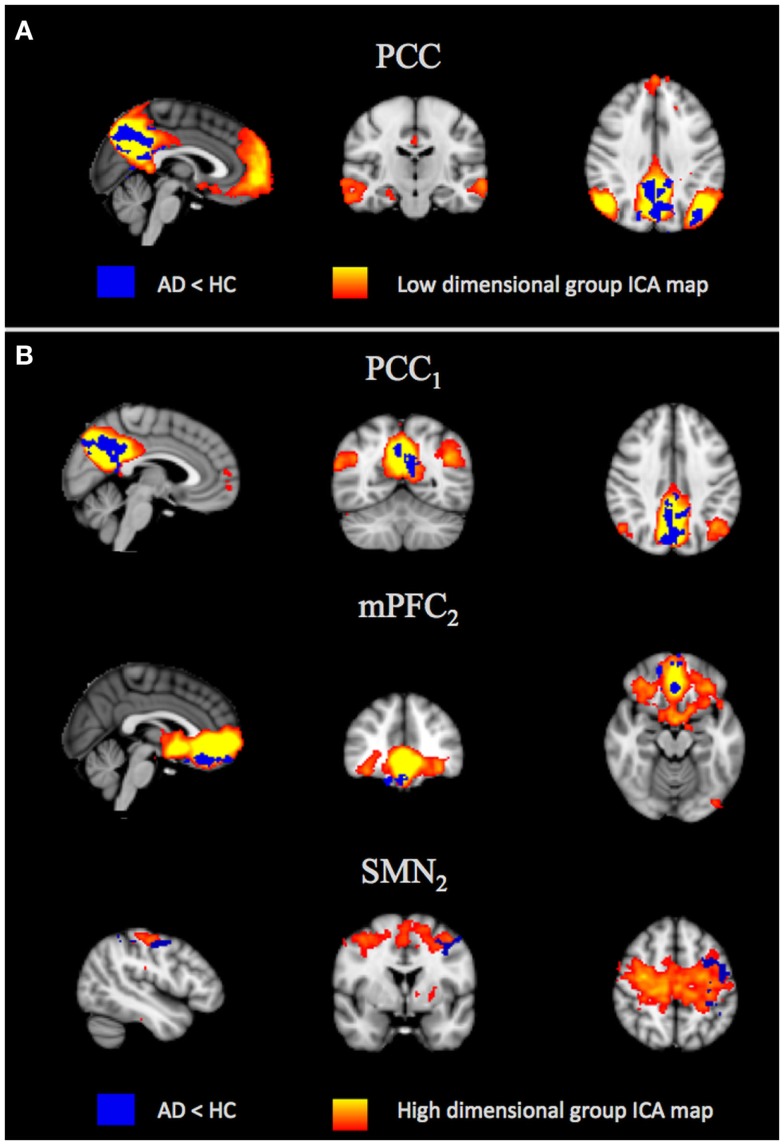
**Between-group differences in resting-state network (RSN) spatial maps**. Group-level ICA spatial maps of the RSNs (red–yellow) at low **(A)** and high **(B)** dimensionality are overlaid with clusters showing significantly lower (blue) functional connectivity in patients with Alzheimer’s disease (AD) relative to healthy controls (HC). Images are shown in radiological orientation. PCC, posterior cingulate cortex; PCC_1_, subnetwork 1 of the PCC; mPFC_2_, subnetwork 2 of the medial prefrontal cortex; SMN_2_, subnetwork 2 of the sensory-motor network.

At low dimensionality, significantly reduced FC was found in patients with AD only in the PCC component, mainly localized in the PCC, the precuneus, and the left superior and inferior parietal lobule (Figure 3A). Significantly lower connectivity in patients with AD was also observed in three high-dimensional components (PCC_1_, mPFC_2_, SMN_2_). In the PCC subnetwork (PCC_1_), the decreased FC was localized in the PCC and the precuneus; the alteration in the mPFC (mPFC_2_) involved the ventral mPFC, while a decreased FC in the SMN was localized in the precentral gyrus (Figure 3B). Results were significant at *p* < 0.05, fully cluster corrected for multiple comparisons after initial cluster-forming thresholding corresponding to *p*_uncorr_ < 0.05. Only the result at low dimensionality survived a further correction across multiple components.

## Discussion

In this work, we applied low-dimensional and high-dimensional group-ICA on rfMRI data of a group of elderly HC and patients with AD in the mild to moderate stage of the disease. The aim of the study was to investigate the effect of the dimensionality of group-ICA decomposition in the detection of FC damage in AD using spatial and temporal (amplitude and network) analyses. The use of an objective and quantitative labeling criterion allowed us to automatically identify the subnetworks of interest and to perform a high-dimensional analysis that was a complementary approach to the low-dimensional one.

We focused the analyses on the DMN (divided in its posterior and anterior portions), as the most damaged by AD (Greicius et al., 2004; Zhang et al., 2009; Gili et al., 2011; Binnewijzend et al., 2012) and on the SMN, for which the role of the pathology is still unclear and under debate (Brier et al., 2012; Damoiseaux et al., 2012).

With regard to the posterior part of the DMN (the PCC), through the low- and high-dimensional spatial map analyses, we verified the loss of activation in the PCC, typical of patients with AD, and extensively supported by literature (Greicius et al., 2004; Zhang et al., 2009; Gili et al., 2011; Binnewijzend et al., 2012). However, thanks to the high-dimensional ICA analysis, we were able to better localize these alterations within the PCC subregions. Moreover, we found a significant decrease in the time series amplitude of both the low- and high-dimensional PCC components of patients with AD, probably because the two PCC subcomponents are equally (and fully) altered in patients with AD with respect to the HC. Interestingly, the reduced amplitude in both the subnetworks does not necessarily imply a FC loss. In fact, the two parts of the posterior DMN, although split into two subnetworks, are anatomically close and can share part of the signal source (i.e., their time series can be highly temporally correlated, as highlighted by their high *z*-value in Figures 2A,B). Hence, our results of the within-network FC at high dimensionality could be explained through a residual short-range FC between the two subnetworks of the posterior DMN in these stages of AD.

With regard to the anterior part of the DMN (the mPFC), no differences in the spatial maps between HC and AD were found at low dimensionality, whereas the same analysis at a higher dimensionality drew attention to a reduced FC in the ventral mPFC subnetwork in AD. This is an interesting result in the light of the time series analysis, which showed decreased amplitude in AD already at low dimensionality. Moreover, the high-dimensional ICA made a significant contribution toward exploring the within-network FC and underlining that the damage could not involve the whole network in the mild to moderate stages of AD, but only the connections between the ventral mPFC component and one of the other two mPFC subnetworks. The alteration in mPFC in AD, described in the advanced stage of AD (Greicius et al., 2004; Zhang et al., 2009, 2010; Gili et al., 2011; Brier et al., 2012; Damoiseaux et al., 2012), has already been correlated to the progression of the structural changes of this pathology (Minoshima et al., 1997; Buckner et al., 2005). We also hypothesize that the initial stages of the disease are affected from mPFC alteration, even if the deficit is not severe enough to be detectable by means of the low-dimensional analysis of spatial maps (Abou Elseoud et al., 2011).

With regard to the SMN, it confirmed to be less altered in patients with AD with respect to the DMN, as evidenced by the high-dimensional ICA analysis of the spatial maps, that showed a decreased FC in only one of the five subnetworks, and from the amplitude analysis, where alterations were observable in the low dimensionality. Interestingly, half of the within-network correlations were significantly lower in patients with AD. We, therefore, hypothesize that the connectivity damage in AD could not be confined to the DMN, but could extend to other areas as the sensory-motor regions [in line with recent findings by Damoiseaux et al. (2012), for patients with AD in a moderate to severe stage], manifesting, in the initial stage of the disease, as a loss of within-network connectivity. This hypothesis is in line with those studies, which demonstrated that motor deficits appear since the disease onset (Scarmeas et al., 2004) and that early motor deficits are associated with a worse disease progression (Scarmeas et al., 2005).

A wider analysis using different ICA dimensionalities would also be useful to define the most suitable model order for the detection of AD alterations. As already pointed out by Abou Elseoud et al. (2011), the higher model order provides higher sensitivity, but also increases the risk of false positives and advanced statistical methods applied at the level of RSNs would be beneficial in order to correct for type I errors. In this context, high-dimensional ICA could be used as a *post hoc* analysis for those networks that show a significant difference between groups at conventional low-dimensional ICA.

Certainly, future studies including subjects in the prodromal stage of AD (mild cognitive impairment) and moderate to severe patients with AD, or longitudinal studies on patients with AD would better clarify whether the changes we observed with the temporal analyses in mild to moderate AD were early signs that anticipate future changes in the spatial maps.

In conclusion, the results herein support the hypothesis that high-dimensional ICA, supported by a component classification based on low-dimensional ICA, can be applied in rfMRI to gain additional knowledge regarding brain FC in applications to AD. A detailed parcellation of the brain and the analysis of the temporal information (e.g., amplitude and network) could give further insight into the detection of FC alterations in pathological conditions and their monitoring at different stages. These promising, albeit preliminary, results obtained in describing the functional disconnections due to this neurodegenerative disease support future developments in this direction.

## Conflict of Interest Statement

The authors declare that the research was conducted in the absence of any commercial or financial relationships that could be construed as a potential conflict of interest.
